# Generative AI to Foster Computational Thinking in Initial Teacher Education: A Thematic Literature Review and Model

**DOI:** 10.3390/bs16040575

**Published:** 2026-04-11

**Authors:** Edwin Creely

**Affiliations:** Faculty of Education, Monash University, Melbourne 3800, Australia; edwin.creely@monash.edu

**Keywords:** computational thinking, generative artificial intelligence, initial teacher education, preservice teachers, self-efficacy, affective engagement, cognitive load, prompt engineering

## Abstract

Computational thinking (CT) has become a cross-curriculum priority in many educational jurisdictions, yet a growing body of research reports uneven integration in initial teacher education (ITE), limited preservice teacher confidence, and persistent misconceptions that equate CT with coding. Concurrently, generative artificial intelligence (GenAI) has rapidly entered university programmes, offering new possibilities for modelling problem-solving, generating multiple representations, and supporting iterative design. However, while constructs such as self-efficacy, cognitive load, and affect are well established in educational psychology, their specific application to the intersection of CT and GenAI in teacher education remains under-theorised: existing research has not systematically examined how these psychological dimensions interact when preservice teachers learn CT through GenAI-mediated tasks. This thematic literature review synthesises 54 sources across three intersecting domains: CT frameworks and their pedagogical implications, CT integration in preservice teacher preparation, and GenAI in teacher education and learning design. Drawing on Bandura’s social cognitive theory, cognitive load theory, and research on technology-related affect, the review foregrounds the affective, cognitive, and cultural dimensions of preservice teachers’ engagement with CT and GenAI. The review proposes the GenAI-Enabled Computational Thinking for Preservice Teachers (GECT-P) model, which integrates CT dimensions with GenAI-supported learning cycles, psychological mediators, and teacher education outcomes. The model positions prompting as an epistemic and pedagogical practice that can make CT visible, supports cycles of decomposition, abstraction, pattern recognition, and algorithmic design, and embeds critical AI literacy, ethics, affective scaffolding, and classroom enactment. Design principles and practical pathways are offered for teacher educators seeking to prepare graduates who can develop CT with and beyond GenAI across diverse curriculum areas.

## 1. Introduction

Computational thinking has emerged as one of the central educational aspirations of the twenty-first century. Since Wing’s (2006) influential articulation of CT as a universally applicable approach to problem formulation and solution design, curriculum frameworks across diverse jurisdictions, including Australia, the United Kingdom, the United States, and several European nations, have positioned CT as a cross-curriculum capability that extends well beyond the boundaries of computer science (Grover & Pea, 2013; Angeli et al., 2016). At its core, CT involves a constellation of reasoning processes: decomposition, abstraction, pattern recognition, and algorithmic thinking, all of which enable learners to formulate problems in ways that computational agents, both human and machine, can address (Wing, 2006; Selby & Woollard, 2013). The ambition is compelling: equip all learners with the intellectual tools to navigate an increasingly data-rich, computationally mediated world.

However, the success of this ambition in schooling depends heavily on teacher capability, and here, the literature reveals a persistent gap. Preservice teacher preparation for CT remains uneven, characterised by narrow conceptualisations that conflate CT with coding, limited curriculum time, and insufficient opportunities for pedagogical enactment across subject areas (Yadav et al., 2014; Listiaji & Molnár, 2025; Rodrigues et al., 2024). At the same time, generative AI technologies that include large language models such as ChatGPT, Claude, and Gemini have entered classrooms and university programmes with remarkable speed, reshaping how learners access information, compose texts, prototype ideas, and engage with complex problems (Giannakos et al., 2024; Ng et al., 2021). These twin developments, the curricular imperative for CT and the disruptive arrival of GenAI, converge in initial teacher education (ITE), raising a practical and theoretically significant question: how can GenAI be integrated into ITE so that it strengthens, rather than replaces, the development of CT knowledge, practices, and dispositions among preservice teachers?

Importantly, this question cannot be addressed through a purely technical or curricular lens. The psychological dimensions of engagement with CT and emerging technologies—the affective, cognitive, and cultural layers of learning—play a decisive role in determining whether preservice teachers develop genuine competence or merely perform surface-level compliance. Research on self-efficacy (Bandura, 1997) demonstrates that teachers’ beliefs about their capability to teach CT significantly influence their willingness to integrate it into classroom practice (Kaya et al., 2020; Rich et al., 2019). Affective responses, including anxiety about technology, curiosity toward novel technologies, and the emotional valence of success and failure during iterative design, shape the depth and persistence of engagement (Burić & Macuka, 2018). Cognitive load theory (Sweller, 1988) alerts us to the risk that poorly designed GenAI tasks may overwhelm working memory rather than scaffold it, particularly for novice learners navigating both CT concepts and unfamiliar AI interfaces simultaneously. Cultural factors, including disciplinary identity, beliefs about what counts as legitimate knowledge, and the norms of professional communities, can mediate how preservice teachers position themselves in relation to both CT and emerging technologies (Ertmer & Ottenbreit-Leftwich, 2010; Tondeur et al., 2012).

The central proposition guiding this article is that GenAI-enhanced CT learning in ITE will be most effective when it is designed to simultaneously support cognitive engagement, build self-efficacy, manage affective responses, and foster critical dispositions toward AI use. Without deliberate attention to these psychological dimensions, there is a significant risk that GenAI integration will reproduce the surface-level engagement and fragmented understanding that have characterised many earlier technology-integration initiatives in teacher education.

This thematic literature review addresses these intersecting concerns by synthesising key strands of research and proposing a model for preservice teacher learning that explicitly foregrounds the psychological dimensions of engagement. The review is organised around five interconnected aims to (a) clarify the definitional landscape of CT and its pedagogical implications for teacher education; (b) examine the current state of CT integration in ITE, with attention to the affective and cognitive challenges preservice teachers face; (c) critically review emerging scholarship on GenAI in teacher education, particularly its psychological affordances and risks; (d) explore how GenAI can serve as a scaffold for CT development when psychological mediators are deliberately addressed; and (e) propose the GenAI-Enabled Computational Thinking for Preservice Teachers (GECT-P) model, which integrates CT dimensions, GenAI-supported learning cycles, psychological scaffolding, and teacher education outcomes.

While previous studies have examined CT in teacher education (Yadav et al., 2014; Rodrigues et al., 2024; Listiaji & Molnár, 2025) or GenAI in educational settings independently (Giannakos et al., 2024; Kasneci et al., 2023), this review makes a distinctive contribution by integrating these two domains with an explicit focus on psychological mediators—self-efficacy, affective engagement, cognitive load, and professional identity—that shape preservice teachers’ capacity to learn and teach CT with GenAI. Existing reviews have focused on curricular structures and cognitive outcomes without systematically theorising the affective and motivational dimensions that determine whether CT knowledge translates into sustained classroom practice. The GECT-P model addresses this gap.

## 2. Materials and Methods

### 2.1. Methodological Approach

This study employs a reflexive thematic literature review methodology, which is particularly suited to synthesising diverse bodies of scholarship around an emerging and interdisciplinary topic (Braun & Clarke, 2006; Snyder, 2019). Unlike a systematic review with predetermined inclusion and exclusion criteria applied to a single research question, a thematic review seeks to identify, analyse, and report patterns across a defined body of literature to generate conceptual insights and, in this case, to inform model development for programme design in ITE. The approach draws on the principles of conceptual synthesis as described by Snyder (2019), who notes that such reviews are particularly appropriate for areas undergoing rapid theoretical development or requiring integration across traditionally separate fields. The intention is not an exhaustive systematic review, but a rigorous conceptual synthesis oriented to identifying themes, tensions, and design implications at the intersection of CT, GenAI, and teacher education.

The thematic review was chosen because the research question spans three intersecting domains unsuited to a single PICO-style question. Following Snyder’s (2019) typology, this approach synthesises scholarship across domains to generate conceptual frameworks rather than aggregate effect sizes. The process was rigorous: a priori search terms with Boolean combinations, five databases searched systematically, explicit inclusion/exclusion criteria, and Braun and Clarke’s (2006) six-phase thematic analysis.

### 2.2. Search Strategy and Platforms

The literature search was conducted from December 2024 to January 2025 across multiple academic databases: Scopus, Web of Science, Education Resources Information Center (ERIC), PsycINFO, and Google Scholar. These databases were selected to ensure coverage of education, educational technology, computer science education, and psychology studies. Scopus and Web of Science provided access to high-impact, peer-reviewed journals across disciplines. ERIC offered comprehensive coverage of education-specific research, including grey literature and conference proceedings relevant to teacher education. PsycINFO was included specifically to capture research on self-efficacy, affective engagement, cognitive load, and technology-related anxiety in educational contexts. Google Scholar was used as a supplementary source for identifying seminal and highly cited works, recent preprints, and works not indexed in other databases.

### 2.3. Search Terms and Criteria

The search strategy employed a combination of terms across three thematic domains, connected using Boolean operators. Table 1 presents the search terms and their combinations.

Searches were limited to English-language publications from 2006 (the year of Wing’s seminal CT article) to January 2025, with particular emphasis on publications from 2018 onwards to capture the most recent developments in GenAI and CT pedagogy. The inclusion criteria required that articles (a) addressed computational thinking in an educational context, (b) focused on teacher education, preservice teacher learning, or pedagogical design for CT, (c) examined generative AI in educational settings, or (d) investigated psychological dimensions (self-efficacy, affect, cognitive load, identity) related to technology integration in teacher education. Articles were excluded if they focused exclusively on K–12 student outcomes without reference to teacher preparation, addressed AI in education without relevance to CT or teacher learning, or were not available in English.

### 2.4. Article Selection and Thematic Analysis

The initial search yielded 847 records across the five databases. After removing duplicates (*n* = 213), the titles and abstracts of 634 records were screened for relevance. Of these, 178 articles were examined in a full-text review. Following full-text assessment against the inclusion criteria, 54 articles were selected for inclusion in the final synthesis. These comprised empirical studies (*n* = 24), systematic and literature reviews (*n* = 12), conceptual and framework papers (*n* = 13), and policy or commentary pieces (*n* = 5). Thematic analysis followed the six-phase approach described by Braun and Clarke (2006): familiarisation with the data, generation of initial codes, searching for themes, reviewing themes, defining and naming themes, and producing the report.

The analysis identified five overarching themes: (a) definitional and conceptual frameworks for CT; (b) the state of CT in ITE; (c) GenAI affordances and risks in teacher education; (d) psychological mediators of engagement with CT and GenAI; and (e) design principles for integrated programmes. These themes structure the results sections that follow and are presented discursively to create a synthesis of the literature that identifies both convergences and tensions across the three domains under review.

To operationalise Braun and Clarke’s (2006) six phases: familiarisation involved repeated reading and annotation of all 54 sources; initial coding was conducted by the sole author using a combination of deductive codes derived from the theoretical framework (self-efficacy, cognitive load, affect, professional identity) and inductive codes that emerged from the data (e.g., false mastery, prompting as epistemic practice); themes were generated by clustering codes into candidate themes, reviewed against the coded extracts and the full dataset, and refined through iterative definition. No specialist qualitative software was used; coding was managed through structured spreadsheets. As a sole-authored thematic review oriented toward conceptual synthesis rather than empirical content analysis, inter-coder reliability testing was not undertaken, consistent with Braun and Clarke’s (2006) reflexive thematic analysis approach, which positions coding as an interpretive rather than reliability-driven process. Included studies were not subjected to formal methodological quality appraisal, as the purpose was conceptual synthesis across diverse study types rather than aggregation of empirical effect sizes; this is acknowledged as a limitation in Section 2.5. The 54 included sources comprised empirical studies (*n* = 24), reviews (*n* = 12), conceptual and framework papers (*n* = 13), and policy or commentary pieces (*n* = 5), spanning CT frameworks, CT in ITE, GenAI in education, and psychological dimensions of technology engagement.

In sum, this is a reflexive thematic analysis study, and thus coding is interpretive and theory-driven, so no inter-coder agreement was sought, which is well aligned with Braun and Clarke’s (2006) argument that rigour should be based not on convergence of evidence but on reflection in the coding process. Additionally, no formal quality appraisal was undertaken, as the purpose of the review was to conduct a conceptual synthesis across different sets of literature, rather than an evaluation or aggregation of empirical effect sizes, and this approach is consistent with the general methodology adopted in integrative and thematic reviews.

Geographically, the included studies were drawn primarily from North America (*n* = 18), Europe (*n* = 14), East Asia (*n* = 9), Australasia (*n* = 7), and multi-national or unspecified contexts (*n* = 6). Educational levels represented included higher education and ITE settings (*n* = 38), primary/elementary education contexts referenced through teacher preparation studies (*n* = 10), and secondary education contexts (*n* = 6). Regarding theme-to-study distribution, Theme (a) on CT definitional frameworks drew primarily on foundational and conceptual sources (Wing, 2006; Brennan & Resnick, 2012; Weintrop et al., 2016; Grover & Pea, 2013; Shute et al., 2017); Theme (b) on CT in ITE was informed by empirical and review studies (Yadav et al., 2014, 2017; Rodrigues et al., 2024; Listiaji & Molnár, 2025; Mouza et al., 2017; Rich et al., 2019); Theme (c) on GenAI in education drew on recent empirical and commentary sources (Giannakos et al., 2024; Kasneci et al., 2023; Bae et al., 2024; Li et al., 2025; Celik et al., 2026); Theme (d) on psychological mediators drew on both domain-specific and general psychology sources (Bandura, 1997; Sweller et al., 2019; Pekrun, 2006; Kaya et al., 2020); and Theme (e) on design principles was synthesised across all four preceding themes. A supplementary table providing a full study-by-theme mapping and summary of included study characteristics is provided as Supplementary Material.

### 2.5. Limitations of the Review

Several limitations should be acknowledged. First, as a thematic and synthetic (rather than systematic) review, the study does not claim exhaustive coverage of all relevant literature. Second, the GenAI landscape is evolving with exceptional rapidity; scholarship published after January 2025 was not included. Third, the psychological dimensions examined (self-efficacy, affect, cognitive load, and professional identity) represent a selective rather than comprehensive treatment of the relevant psychology literature. Finally, the proposed GECT-P model is conceptual and has not yet been empirically tested; it is offered as a design framework to guide future intervention research and practice contexts.

## 3. Results

The Introduction established the broad theoretical rationale for attending to psychological dimensions in CT and GenAI integration, drawing on social cognitive theory, cognitive load theory, and research on technology-related affect to frame the central proposition of this article. The results that follow move beyond this theoretical framing to present the specific thematic findings that emerged from the synthesis of the 54 reviewed sources. Where foundational constructs such as self-efficacy or cognitive load reappear, they do so not as reiterated theory but as empirically grounded patterns identified across the reviewed studies, with attention to how they manifest specifically in preservice teachers’ engagement with CT and GenAI in ITE contexts.

### 3.1. Computational Thinking: Definitions, Dimensions, and Psychological Underpinnings

Wing’s (2006) framing of CT as a way of thinking that involves formulating problems and expressing solutions in forms that a computational agent can carry out has been foundational, but the subsequent proliferation of definitions has created what Grover and Pea (2013) describe as a “cacophony” of competing conceptualisations. Educational work has progressively stressed that CT is not limited to programming; rather, it involves transferable ways of reasoning that include decomposition, abstraction, pattern recognition, and algorithmic thinking, as well as practices of iterative design, debugging, and testing (Barr & Stephenson, 2011; Shute et al., 2017; Selby & Woollard, 2013). Brennan and Resnick’s (2012) three-dimensional framework is especially useful for teacher education because it positions CT as comprising (a) concepts, (b) practices, and (c) perspectives, where perspectives include dispositions such as seeing oneself as a creator and recognising the social and expressive dimensions of computing. Similarly, Weintrop et al. (2016) offer a taxonomy of CT practices for mathematics and science that highlights data practices, modelling and simulation, computational problem-solving, and systems thinking, thereby extending the conceptual repertoire available to teacher educators.

What is often under-represented in purely cognitive accounts of CT is the affective and motivational substrate upon which computational reasoning develops. From a psychological perspective, CT engagement involves not merely knowing what decomposition or abstraction entails but experiencing the frustration and satisfaction of iterative debugging, the curiosity that drives pattern seeking, and the confidence required to persist when algorithmic solutions fail. Bandura’s (1997) social cognitive theory provides a useful lens: self-efficacy mediates the relationship between CT knowledge and CT practice. Research by Kaya et al. (2020) confirms that targeted CT interventions can improve preservice elementary teachers’ CT teaching efficacy beliefs, and that these beliefs are distinct from general technology self-efficacy. Yadav et al. (2017) further argue that CT must be explicitly infused into teacher education curricula to develop both conceptual understanding and pedagogical confidence.

Cognitive load theory (Sweller, 1988; Sweller et al., 2019) offers a complementary perspective on the cognitive demands of CT learning. CT tasks inherently carry significant intrinsic cognitive load because they require simultaneous attention to problem structure, representational abstraction, and procedural logic. When CT is combined with unfamiliar technological environments, whether visual programming platforms or GenAI interfaces, extraneous load can escalate rapidly, consuming working memory resources that should be devoted to germane processing. For preservice teachers who are simultaneously learning CT concepts, navigating pedagogical reasoning, and managing new tools, the cognitive burden can be substantial (Kalyuga, 2011). This suggests that instructional design for CT in ITE must deliberately attend to load management, a point that becomes even more critical when GenAI is introduced as a mediating technology.

Cultural dimensions of CT engagement also warrant attention. Kafai and Proctor (2022) argue for a critical computing perspective that situates CT within broader questions of power, equity, and cultural relevance. For preservice teachers preparing to work in diverse classrooms, CT cannot be treated as a culturally neutral skill set. The examples, contexts, and problems through which CT is taught carry cultural assumptions about whose knowledge counts and what problems are worth solving. This cultural dimension intersects with psychological constructs: preservice teachers from non-STEM backgrounds may experience disciplinary identity threat when confronted with computing tasks, while those from underrepresented communities may encounter additional barriers related to stereotype threat and belonging uncertainty (Cheryan et al., 2017). A psychologically informed approach to CT in ITE must therefore attend to the cultural work of creating inclusive learning environments where all preservice teachers can develop CT confidence and competence regardless of their disciplinary background or prior computing experience.

### 3.2. Computational Thinking in Initial Teacher Education: The Affective and Cognitive Landscape

Research consistently indicates that preservice teachers often hold narrow views of CT, lack confidence to teach it, and struggle to recognise CT opportunities outside STEM contexts. Yadav et al. (2014) provided early and influential evidence that targeted modules in teacher education can improve preservice teachers’ understanding of CT concepts and attitudes towards computing. More recent systematic reviews offer a broader picture. Rodrigues et al. (2024) synthesise primary teacher education studies and show that CT integration varies widely in duration, structure, and assessment, with many interventions consisting of brief, isolated workshops rather than sustained, embedded experiences. Listiaji and Molnár (2025) similarly report that research is concentrated in preservice STEM settings, that visual programming and plugged activities dominate, and that questionnaires and achievement tests are the most common assessment measures. Mouza et al. (2017) found that integrating CT into an educational technology course for preservice teachers improved understanding but required careful scaffolding to bridge the gap between personal competence and pedagogical application.

Across this literature, several psychological challenges recur with striking regularity. The first is the persistent “coding equals CT” misconception, which functions not merely as a definitional error but as an affective barrier: preservice teachers who believe CT is synonymous with programming frequently report anxiety, low confidence, and avoidance behaviours that constrain their engagement (Bower et al., 2017; Yadav et al., 2014). The second challenge is limited self-efficacy for CT teaching, particularly among preservice teachers in non-STEM disciplines. Rich et al. (2019) found that elementary preservice teachers’ CT self-efficacy was significantly predicted by prior computing experience. Sentance and Csizmadia (2017) similarly document how secondary teachers struggle with confidence when expected to teach computing concepts for the first time. The third challenge is the difficulty of pedagogical transfer: even when preservice teachers develop personal CT skills, translating these into classroom practice across subjects requires a further act of pedagogical imagination that is itself cognitively demanding and emotionally risky (Liu et al., 2024; Angeli & Valanides, 2020).

Where interventions are more successful in addressing these psychological dimensions, they tend to share common features: explicit connections to curriculum content that validate non-STEM applications of CT; modelling of CT pedagogy by teacher educators who demonstrate both competence and vulnerability; opportunities for micro-teaching or lesson design that build mastery experiences (the most potent source of self-efficacy, per Bandura, 1997); and reflective work that helps preservice teachers articulate how CT can be enacted with learners in emotionally safe and culturally responsive ways. These findings suggest that the quality of CT learning in ITE is as much a function of psychological and relational design as it is of curricular content (Hsu et al., 2018). Importantly, these psychological dimensions are not merely obstacles to be overcome but constitutive features of professional learning that, when properly addressed, can deepen rather than constrain the development of CT competence and pedagogical expertise.

### 3.3. Generative AI in Education and Teacher Preparation: Affordances, Risks, and Psychological Dimensions

Generative AI has been positioned as both an opportunity and a disruption in education. Within the broader educational technology landscape, GenAI models, bots, and platforms represent a qualitative shift from earlier technologies because they can generate novel content, engage in dialogue, and produce outputs that simulate expert reasoning across domains. Commentaries note its capacity to support learning design, feedback, differentiation, and iterative refinement of work, while also raising substantive concerns about integrity, bias, transparency, dependency, and the erosion of critical thinking (Giannakos et al., 2024; Baidoo-Anu & Ansah, 2023; Kasneci et al., 2023). Within teacher education specifically, studies report mixed preservice teacher views that combine optimism about efficiency and creativity with uncertainty about ethics, accuracy, and assessment implications. Panday-Shukla (2025) reports that preservice teachers and teacher educators may display moderate digital literacy while AI literacy remains comparatively low. Moorhouse et al. (2023) highlight teacher educators’ own uncertainties about GenAI, noting that institutional responses range from outright prohibition to tentative experimentation.

The psychological dimensions of preservice teachers’ engagement with GenAI are particularly revealing. Recent research has applied technology acceptance frameworks, including the Technology Acceptance Model and the Unified Theory of Acceptance and Use of Technology, to examine the cognitive and affective factors that shape GenAI adoption. Findings consistently highlight the role of perceived usefulness and self-efficacy as positive predictors of behavioural intention to use GenAI, while technology anxiety and concerns about academic integrity function as significant inhibitors (Li et al., 2025; Konca et al., 2025). Personality traits such as openness to experience and conscientiousness have been found to moderate acceptance (Li et al., 2025). Chiu (2021) proposes a framework for teacher AI competence that extends TPACK to include AI-specific dimensions, arguing that teacher preparation must develop not just technical skills but also adaptive expertise in working with AI systems.

From a cognitive load perspective, GenAI introduces both opportunities and risks. GenAI can function as an external cognitive resource that offloads certain processing demands, for instance, by generating initial code structures, providing worked examples, or summarising complex texts, thereby freeing working memory for higher-order reasoning. On the other hand, the opacity of GenAI outputs, the need to evaluate AI-generated content critically, and the simultaneous management of prompting strategies and domain knowledge can create substantial extraneous load, particularly for novice users (Sweller et al., 2019; Kalyuga, 2011). This dual cognitive character of GenAI, namely, its capacity to simultaneously reduce and increase cognitive load depending on task design, has not been sufficiently theorised in the teacher education literature. Accordingly, I argue that cognitive load management should be a central design consideration in GenAI-enhanced CT learning, and that task designers must carefully calibrate the balance between offloading routine processing and maintaining the effortful engagement that builds durable understanding.

Research on prompting competency with GenAI in teacher education suggests that prompt engineering is not merely a technical skill but an epistemic and pedagogical practice shaped by teachers’ professional knowledge. Celik et al. (2026) demonstrate that higher-quality prompting strategies are associated with more adaptive lesson plan outputs and are mediated by AI-specific technological and pedagogical knowledge, which they conceptualise as Intelligent-TPACK. Park and Choo (2025) provide practical strategies for educator prompting, reinforcing that prompting can be taught as a structured practice. Deng and Yu (2023) argue that prompt literacy should be considered a core competency within AI literacy frameworks, positioning the ability to formulate effective prompts as a form of computational expression. It is important to note that these terms carry distinct implications: prompt engineering refers to the technical skill of crafting effective inputs; prompt literacy denotes a broader understanding of how prompts shape AI outputs, akin to a critical literacy; and prompting as epistemic practice positions the act of prompting as a form of inquiry and knowledge construction. This review uses prompting as the encompassing term and specifies the narrower constructs where precision is required.

The affective landscape of preservice teachers’ GenAI engagement also warrants careful attention. Preservice teachers report complex mixtures of excitement about creative possibilities, anxiety about professional replacement, guilt about potential over-reliance, and frustration when outputs fail to meet expectations (Bae et al., 2024). From the perspective of Pekrun’s (2006) control-value theory, each emotion reflects a particular configuration of perceived control and perceived value. Moreover, the cultural context of GenAI adoption in teacher education cannot be overlooked. Institutional norms, disciplinary traditions, and professional cultures shape how preservice teachers interpret and respond to GenAI tools (Bae et al., 2024). In contexts where academic integrity discourses frame AI use primarily as cheating, preservice teachers may experience shame or moral conflict that inhibits productive engagement.

### 3.4. GenAI as a Scaffold for Computational Thinking

The convergence of CT and GenAI in teacher education opens productive possibilities, but only when the psychological conditions for meaningful learning are deliberately cultivated. Four primary affordances of GenAI for CT development are identified, each analysed through an affective, cognitive, and cultural lens.

#### 3.4.1. Externalisation of Reasoning

GenAI can produce worked examples, alternative solutions, and explanatory narratives that preservice teachers can critique, compare, and evaluate. From a cognitive load perspective, this externalisation reduces the intrinsic load of simultaneously generating and evaluating solutions, allowing learners to focus cognitive resources on analysis and judgement (Ainsworth, 2006). Affectively, the availability of AI-generated examples can reduce the anxiety associated with a blank starting point. However, if GenAI outputs are accepted uncritically, the externalisation becomes a substitution that bypasses rather than supports reasoning (Liao et al., 2024; Tedre et al., 2021).

#### 3.4.2. Support for Iteration

GenAI enables rapid prototyping, debugging, and revision cycles that align with CT practices of testing, evaluating, and refining solutions. Psychologically, iteration is where self-efficacy is built. Bandura (1997) identifies mastery experiences as the most potent source of efficacy beliefs. The key design consideration is that iteration must involve genuine cognitive engagement, not merely regenerating prompts until a satisfactory output appears, but systematically diagnosing errors, modifying constraints, and testing hypotheses about why outputs succeed or fail.

#### 3.4.3. Multiple Representations

GenAI can generate diverse representations of the same problem—textual, visual, algorithmic, and narrative—supporting the abstraction and pattern recognition central to CT. From a cognitive science perspective, multiple representations facilitate transfer by helping learners identify structural features that transcend surface characteristics (Ainsworth, 2006). Culturally, the capacity to generate representations in different disciplinary contexts supports the cross-curricular CT integration that curriculum frameworks envision (Israel et al., 2015).

#### 3.4.4. Contextualisation Across Disciplines

GenAI can adapt CT tasks to different curriculum areas such as mathematics, literacy, science, the arts, and social studies, allowing preservice teachers to see CT as genuinely cross-disciplinary. This affordance directly addresses the cultural dimension of CT engagement: by demonstrating that CT is not the exclusive province of STEM, it challenges the disciplinary identity barriers that constrain engagement among preservice teachers in humanities and arts specialisations (Ertmer & Ottenbreit-Leftwich, 2010; Mishra & Koehler, 2006).

These affordances are emphatically not automatic. If GenAI is used as a shortcut, it can obscure the very reasoning processes CT seeks to develop. Concerns about false mastery and over-reliance have been raised (Giannakos et al., 2024; Kasneci et al., 2023; Zastudil et al., 2023), suggesting that learners may appear competent while relying on AI outputs without understanding underlying concepts. From a psychological perspective, false mastery is particularly insidious because it can inflate self-efficacy without grounding it in genuine capability. A related and emerging concern is AI dependency, where frequent reliance on GenAI tools may weaken autonomous cognition. Recent research has shown that generative AI dependency is associated with cognitive failures in both younger and older adults (Goh et al., 2025), suggesting that habitual offloading of reasoning to AI systems may erode the very cognitive capacities that CT education seeks to develop. This risk is particularly salient in ITE, where preservice teachers must build robust independent reasoning alongside technology-mediated skills. Therefore, GenAI-enhanced CT learning in ITE requires deliberate design that makes thinking visible, requires explanation and justification, and builds verification and reflection into every task. The critical question is not whether GenAI should be used in CT education but how it can be used in ways that preserve and strengthen the cognitive engagement upon which genuine understanding depends.

### 3.5. Toward an Integrated Model: Psychological Mediators in GenAI-Enhanced CT Learning

The thematic review reveals that the relationship between GenAI use and CT development is mediated by multiple psychological factors that existing models inadequately address. Teacher knowledge frameworks such as TPACK (Mishra & Koehler, 2006) capture the intersection of technological, pedagogical, and content knowledge but do not explicitly theorise the affective and motivational dimensions that determine whether knowledge is translated into practice. Similarly, CT frameworks (Brennan & Resnick, 2012; Weintrop et al., 2016) define the cognitive dimensions of CT but give limited attention to the emotional, relational, and cultural conditions under which CT learning occurs. An integrated model for GenAI-enhanced CT in ITE must account for at least four psychological mediators that operate across individual, relational, and cultural levels of the learning experience.

#### 3.5.1. Self-Efficacy for CT and GenAI Use

Drawing on Bandura (1997), this construct encompasses preservice teachers’ confidence in their ability to understand CT concepts, apply CT practices, teach CT in classroom contexts, and use GenAI tools productively. Interventions should target CT-specific efficacy through graduated mastery experiences, vicarious observation, verbal encouragement, and attention to physiological and affective states during CT tasks (Kaya et al., 2020).

#### 3.5.2. Affective Engagement and Regulation

Preservice teachers’ emotional responses to CT and GenAI—anxiety, frustration, curiosity, satisfaction, excitement—shape the quality and persistence of their engagement. Research on teachers’ emotions demonstrates bidirectional relationships between affect and self-efficacy (Burić et al., 2020) and between affect and cognitive processing (Pekrun, 2006). GenAI-enhanced CT tasks should be designed to provide a sense of agency while connecting to preservice teachers’ professional goals.

#### 3.5.3. Cognitive Load Management

Effective GenAI-enhanced CT learning requires careful attention to the balance between intrinsic, extraneous, and germane cognitive load (Sweller et al., 2019). Task design should leverage GenAI to reduce extraneous load through scaffolded prompts, worked examples, and structured output formats while maintaining sufficient intrinsic load for meaningful learning and actively promoting germane load through reflection, explanation, and comparison activities.

#### 3.5.4. Professional Identity and Critical Disposition

How preservice teachers see themselves in relation to CT and GenAI—whether as consumers, critics, or creators—constitutes a dimension of professional identity that shapes long-term practice. Developing a critical disposition toward GenAI—what might be termed critical AI literacy (Long & Magerko, 2020; Ng et al., 2021)—requires not merely knowledge of AI limitations but a professional identity that positions responsible, ethical use as central to good teaching.

## 4. Discussion

Synthesising across the CT, GenAI, and psychology literatures, this section first identifies critical tensions in the reviewed findings, then proposes five design principles and a model for ITE programmes that seek to integrate GenAI-enhanced CT learning with explicit attention to psychological dimensions.

The design principles draw on three complementary theories. Bandura’s (1997) social cognitive theory explains self-efficacy formation through mastery experiences and predicts that graduated GenAI-supported tasks will build CT teaching persistence. Cognitive load theory (Sweller et al., 2019) provides the instructional design logic for sequencing tasks so that it scaffolds free working memory for deep processing. Vygotsky’s (1978) zone of proximal development frames GenAI as scaffolded mediation enabling engagement beyond independent capacity, with support fading as competence develops.

Before turning to these design principles, several critical tensions emerging from the reviewed findings warrant discussion. First, the literature reveals a persistent disconnect between how CT is conceptualised in curriculum frameworks and how it is enacted in ITE programmes: while frameworks position CT as cross-curricular and dispositional, most reported interventions remain short-term, tool-focused, and confined to STEM contexts (Rodrigues et al., 2024; Listiaji & Molnár, 2025). Second, the reviewed studies on GenAI in teacher education expose a paradox: the same technology that can scaffold CT reasoning also risks undermining it through uncritical dependency and inflated self-efficacy ungrounded in genuine mastery (Giannakos et al., 2024; Zastudil et al., 2023). Third, the psychological mediators identified in the results—self-efficacy, affect, cognitive load, and professional identity—are treated largely as independent variables across the reviewed literature, yet the findings suggest they interact dynamically: for instance, high cognitive load during GenAI tasks may depress self-efficacy, which in turn heightens anxiety and constrains the very engagement needed to develop CT competence. The design principles and model that follow attempt to address these tensions but should be read as theoretically informed responses to unresolved challenges rather than as settled prescriptions.

### 4.1. Design Principles

Each of the following principles is derived from the thematic synthesis presented in Section 3 and is grounded in specific findings from the reviewed literature, as indicated by the citations accompanying each principle.

*Principle 1: Position CT as cross-curricular and conceptually broader than coding.*

Programmes should challenge the coding–CT conflation not merely through definitional instruction but through affective and identity work that helps preservice teachers from all disciplines see themselves as CT-capable practitioners. This involves providing CT experiences in diverse disciplinary contexts, using GenAI to generate discipline-specific CT problems, and creating peer learning environments where non-STEM and STEM preservice teachers collaborate (Yadav et al., 2014; Rodrigues et al., 2024; Israel et al., 2015).

*Principle 2: Teach prompting as a CT-aligned epistemic practice.*

Prompting should be reconceptualised as an iterative cycle of specifying goals, providing constraints, testing outputs, diagnosing errors, and refining prompts, becoming a process that mirrors the decomposition, algorithmic design, and debugging practices central to CT (Celik et al., 2026; Park & Choo, 2025; Deng & Yu, 2023). Crucially, prompting should be taught as a skill that develops through practice and feedback, building domain-specific self-efficacy through graduated task complexity.

*Principle 3: Design for cognitive load management*.

Tasks should use structured scaffolds (including prompt templates, worked examples of CT reasoning, and progressive complexity) that reduce extraneous load while maintaining cognitively demanding core activities. GenAI should be introduced after foundational CT concepts are established to avoid split attention effects and used to support rather than replace the effortful processing that builds durable schemas (Sweller et al., 2019; Kalyuga, 2011). To clarify the intended developmental sequencing: this principle does not preclude GenAI use with novice learners but recommends that initial CT encounters focus on unplugged or low-technology activities to establish core concepts (decomposition, abstraction, pattern recognition), after which GenAI is introduced as a scaffold that extends rather than replaces foundational understanding. The sequencing is graduated, not binary: as CT competence develops, GenAI tasks increase in complexity and autonomy.

*Principle 4: Embed critical AI literacy with affective scaffolding.*

Accuracy checking, bias awareness, transparency about tool use, and ethical decision-making should be integrated into every GenAI-CT task. Affective scaffolding means creating emotionally safe environments where preservice teachers can express uncertainty about AI, experience productive failure without threat, and develop critical dispositions grounded in professional responsibility rather than technophobia (Long & Magerko, 2020; Kasneci et al., 2023; Ng et al., 2021).

*Principle 5: Include pedagogical enactment with reflective practice.*

Lesson design, micro-teaching, and assessment planning should translate GenAI-supported CT learning into classroom practice. Reflective activities that include design logs, prompt iteration journals, and peer observation protocols provide opportunities for preservice teachers to develop metacognitive awareness and to build the narrative of professional identity that sustains CT integration beyond the ITE programme (Ertmer & Ottenbreit-Leftwich, 2010; Zimmerman, 2002).

### 4.2. Proposed Model: GenAI-Enabled Computational Thinking for Preservice Teachers (GECT-P)

**Figure 1:**
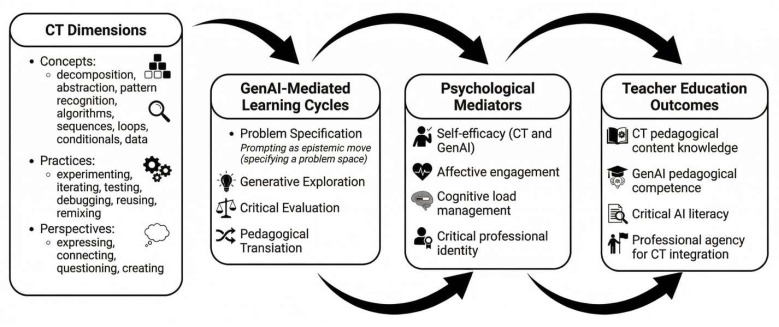
The GenAI-Enabled Computational Thinking for Preservice Teachers (GECT-P).

The GECT-P model (Figure 1) integrates CT dimensions with GenAI-supported learning cycles, psychological mediators, and teacher education outcomes. It is organised into four interrelated layers that reflect the multidimensional nature of the learning it seeks to support.

CT Dimensions (Foundational Knowledge). This layer draws on Brennan and Resnick’s (2012) framework and encompasses three dimensions: CT concepts (decomposition, abstraction, pattern recognition, algorithms, sequences, loops, conditionals, data), CT practices (experimenting, iterating, testing, debugging, reusing, remixing), and CT perspectives (expressing, connecting, questioning, creating).

GenAI-Mediated Learning Cycles (Pedagogical Process). This layer describes the process through which GenAI supports CT learning through four recursive phases: (a) Problem Specification, where preservice teachers decompose a problem and formulate an initial GenAI prompt; (b) Generative Exploration, where GenAI produces outputs that preservice teachers analyse; (c) Critical Evaluation, where outputs are tested against criteria, errors are diagnosed, and prompts are refined; and (d) Pedagogical Translation, where CT insights are translated into lesson plans, teaching resources, and assessment designs. In this model, prompting is treated as an epistemic move that can make CT visible, so that a prompt is not a request for an answer but a specification of a problem space that mirrors algorithmic design.

Psychological Mediators (Enabling Conditions). This layer represents the psychological factors that mediate the effectiveness of the learning cycles: self-efficacy for CT and GenAI (developed through mastery experiences, modelling, and feedback); affective engagement (supported through task design that promotes curiosity, manageable challenge, and emotional safety); cognitive load management (addressed through scaffolding, progressive complexity, and deliberate sequencing); and critical professional identity (fostered through ethical reasoning, transparency practices, and reflective activities). This layer is what distinguishes the GECT-P model from existing CT integration frameworks. It makes explicit the psychological work that must accompany cognitive and technical learning if that learning is to translate into sustained professional practice rather than remaining an isolated competence that atrophies once external supports are removed.

Teacher Education Outcomes (Professional Capability). This layer specifies the intended outcomes: CT pedagogical content knowledge, GenAI pedagogical competence, critical AI literacy, and professional agency for CT integration. The four layers are not hierarchical but recursive and bidirectional. Mastery experiences in the learning cycle build self-efficacy (Layer 3), which enables more ambitious engagement with CT dimensions (Layer 1), which feeds back into more sophisticated prompting and pedagogical translation (Layer 2), which strengthens professional outcomes (Layer 4).

A concrete illustration may clarify how the model operates. Consider a preservice teacher specialising in primary literacy who is asked to design a lesson that integrates CT through a narrative sequencing activity. In Layer 1, the relevant CT concepts are sequencing, decomposition, and algorithmic thinking. In Layer 2, the preservice teacher uses GenAI to generate multiple story outlines with different structural patterns, evaluates which structures best support the learning objective, refines the prompt, and translates the final activity into a lesson plan. In Layer 3, the teacher educator attends to psychological mediators: modelling the prompting process to reduce anxiety, providing a prompt template to manage cognitive load, offering formative feedback that builds confidence, and facilitating peer discussion about the ethical implications of using AI-generated content. In Layer 4, the preservice teacher demonstrates CT pedagogical content knowledge, GenAI pedagogical competence, critical AI literacy, and professional agency.

### 4.3. Implications for Theory and Research

The GECT-P model extends existing frameworks in several theoretically significant ways. First, by explicitly incorporating psychological mediators, the model addresses a gap identified across multiple reviews: the tendency of CT integration research to focus on cognitive outcomes while neglecting the affective and motivational dimensions that determine whether those outcomes translate into sustained practice (Liu et al., 2024; Kaya et al., 2020; Mouza et al., 2017). Second, the model’s treatment of prompting as an epistemic practice connects GenAI use to established traditions in educational psychology around metacognition, self-regulated learning, and epistemic cognition. When preservice teachers iteratively refine prompts, they engage in metacognitive monitoring that parallels the self-regulatory processes identified as central to expert performance (Zimmerman, 2002).

Third, the model’s emphasis on critical professional identity responds to emerging concerns about the cultural and ethical dimensions of AI in education (Long & Magerko, 2020; Kafai & Proctor, 2022; Tedre et al., 2021). By positioning critical AI literacy as an aspect of professional identity, the model suggests that sustainable CT integration requires preservice teachers to develop a professional self that sees ethical reasoning, transparency, and critical evaluation as constitutive of good teaching. For future empirical research, the model generates testable hypotheses: that GenAI-enhanced CT interventions that include explicit self-efficacy scaffolding will produce greater gains in CT teaching confidence; that cognitive load management through structured prompting scaffolds will improve CT learning outcomes; and that affective engagement will mediate the relationship between GenAI task design and CT learning persistence.

It is important to clarify what the GECT-P model explains that existing frameworks do not. TPACK (Mishra & Koehler, 2006) and its AI-extended variants (Chiu, 2021; Celik et al., 2026) map the knowledge domains teachers need but do not theorise the psychological processes through which that knowledge is acquired, sustained, or translated into practice. CT integration frameworks (Brennan & Resnick, 2012; Weintrop et al., 2016) define what CT comprises but give limited attention to the affective, motivational, and identity-based conditions under which CT learning occurs. AI literacy frameworks (Long & Magerko, 2020; Ng et al., 2021) specify competencies for understanding AI but do not connect these to CT pedagogy or teacher professional development. The GECT-P model’s distinctive contribution is that it integrates these domains and adds an explicit psychological mediation layer—self-efficacy, affect, cognitive load, and professional identity—that none of the existing frameworks adequately addresses. In this sense, the model does not replace TPACK, CT, or AI literacy frameworks but positions them within a broader architecture that foregrounds the enabling conditions for their effective enactment in ITE. Thus, the GECT-P model does not seek to expand the knowledge dimensions of TPACK or AI literacy. Rather, it extends TPACK by theorising the psychological processes that mediate how such knowledge is enacted in practice, including self-efficacy, affective engagement, cognitive load, and professional identity, each of which may either facilitate or inhibit a teacher’s capacity to translate knowledge into effective pedagogical action.

The present synthesis also extends recent systematic reviews of AI in education. For instance, Feng and Carolus (2026) review AI literacy at school with a focus on psychological foundations but do not address CT integration or teacher education specifically. Crompton and Burke (2023) provide a broad systematic review of AI in higher education across trends, benefits, and challenges but do not foreground the psychological mediators that shape teacher learning or propose a model for CT-GenAI integration in ITE. The GECT-P model builds on this prior work by connecting AI literacy and CT pedagogy through an explicit psychological mediation layer oriented to preservice teacher professional development. Regarding boundary conditions, the model’s testable hypotheses require specification of moderators: GenAI is likely to reduce cognitive load when tasks are well-structured and learners have foundational CT knowledge, but to increase load when tasks are open-ended and learners lack prior CT experience. Similarly, prior computing experience is expected to moderate the relationship between GenAI use and CT self-efficacy, with novice learners benefiting most from scaffolded prompting sequences and experienced learners benefiting from more autonomous GenAI-mediated inquiry. These boundary conditions should be operationalised in future empirical designs.

### 4.4. Implications for Teacher Educators and Programme Design

Implementing the GECT-P model suggests several actionable steps for ITE programmes. First, programmes can embed short, repeated CT-with-GenAI experiences across multiple units rather than isolating CT in a single subject, providing the spaced practice that supports durable schema construction and the repeated mastery experiences that build self-efficacy (Bandura, 1997; Hsu et al., 2018). Second, assessment can foreground process evidence (design logs, prompt iteration journals, reflective rationales, peer feedback) alongside final products, thereby emphasising the cognitive and affective dimensions of learning that the model identifies as critical. Third, teacher educators can model responsible GenAI use by publicly demonstrating their own prompting processes, sharing their uncertainties, requiring transparency statements, and building verification routines into collaborative tasks (Moorhouse et al., 2023).

Fourth, partnerships with placement schools can support preservice teachers to trial CT-infused lessons and reflect on learner responses, providing the authentic mastery experiences essential for consolidating both CT competence and professional identity. Fifth, attention to the affective environment is not a supplementary concern but a design imperative: teacher educators should explicitly acknowledge the emotional challenges of CT and GenAI learning, normalise productive struggle, and create peer support structures that buffer against the isolation and anxiety that can accompany technology-intensive learning. Finally, cultural responsiveness should guide the selection of CT problems and GenAI tasks, ensuring that examples reflect diverse communities, knowledge systems, and professional contexts (Kafai & Proctor, 2022; Israel et al., 2015).

Three disciplinary examples illustrate practical implementation. In primary mathematics, preservice teachers use GenAI to decompose word problems, generate solution pathways, and design lessons, scaffolding the same CT process for students. In secondary English, they prompt GenAI to generate different narrative outlines and design activities where students create sequencing algorithms for storytelling. In science, they model experimental designs and create worksheets guiding learners through decomposition. Each example enacts the GECT-P model’s four layers.

### 4.5. Limitations of the Study and Model

Several limitations should be acknowledged. The thematic review methodology, while appropriate for interdisciplinary synthesis, does not provide the statistical rigour of meta-analytic approaches. The proposed model is conceptual and has not been empirically validated; its layers and mediators require careful operationalisation and measurement in future research designs that can capture the dynamic and recursive interactions the model proposes. The review’s emphasis on English-language literature may underrepresent important developments in non-Anglophone contexts, particularly in East Asia and Latin America, where CT and AI integration are advancing rapidly. The psychological constructs examined are selective; other relevant constructs—including epistemic beliefs, creative self-efficacy, and collective efficacy among teaching teams—deserve attention in future work. Finally, the rapid evolution of GenAI tools means that specific technological affordances discussed here may shift; the model is intended to be technology-agnostic in its principles.

A further limitation concerns the relative paucity of empirical research that directly examines the intersection of all three domains. While each domain has generated substantial scholarship independently, studies that investigate how psychological mediators operate specifically within GenAI-enhanced CT learning in ITE remain scarce. The GECT-P model is therefore necessarily speculative in some of its mechanisms. This limitation simultaneously highlights the contribution of the model: by making these psychological mechanisms explicit and generating testable hypotheses, it creates a framework that future empirical research can systematically evaluate and refine.

Empirical validation is an essential next step. Future research should adopt mixed-methods designs combining pre-post CT self-efficacy measures (using instruments such as those developed by Kaya et al., 2020), cognitive load surveys, and CT rubrics with think-aloud protocols, reflective journals, and interviews. A phased approach is recommended: pilot studies in single ITE units, quasi-experimental comparisons across disciplines and institutions, and longitudinal research tracking transfer of GenAI-supported CT competence into early-career practice.

## 5. Conclusions

This thematic literature review has examined the intersection of computational thinking, generative artificial intelligence, and the psychology of engagement in initial teacher education. The synthesis of 54 sources spanning nearly two decades of scholarship reveals that CT integration in ITE remains uneven, often constrained by narrow conceptualisations, limited confidence, and insufficient attention to the affective and cognitive dimensions that determine whether knowledge translates into practice. Concurrently, GenAI introduces new affordances for externalising reasoning, supporting iteration, generating multiple representations, and contextualising CT across disciplines, but these affordances carry psychological risks, including false mastery, cognitive overload, and uncritical dependency.

The proposed GECT-P model offers a coherent framework for aligning CT dimensions, GenAI-mediated learning cycles, psychological mediators, and teacher education outcomes. Its distinctive contribution is the explicit theorisation of self-efficacy, affective engagement, cognitive load management, and professional identity as enabling conditions for effective GenAI-enhanced CT learning. The model positions prompting as an iterative, CT-aligned epistemic practice and insists that CT must be enacted as teaching practice, not only as personal skill development.

The broader significance of this work lies in its argument that the affective, cognitive, and cultural dimensions of engagement with computational thinking and emerging technologies are not peripheral considerations but central design imperatives. As teacher education programmes respond to the twin challenges of CT curriculum integration and GenAI disruption, the temptation will be to focus on technical skills and tool proficiency. This review suggests that such a focus, without concurrent attention to the psychological substrate of learning, will produce preservice teachers who can use CT and GenAI technologies but who lack the confidence, critical disposition, and professional identity to integrate them meaningfully into their future classrooms. The GECT-P model offers a pathway beyond this limitation, proposing that the most effective teacher education programmes will be those that treat cognitive challenge, emotional safety, and cultural responsiveness as the foundation upon which technical capability is built.

Future empirical work can test the model’s hypotheses across disciplines and contexts, examine how preservice teachers transfer GenAI-supported CT learning into classroom practice, and investigate the longitudinal development of CT self-efficacy and professional identity in the early years of teaching. In doing so, such research will contribute not only to the fields of CT education and AI in education but also to the broader project of understanding how human engagement with emerging technologies is shaped by the intricate interplay of thinking, feeling, and belonging.

## Figures and Tables

**Table 1:** Search Terms and Boolean Combinations.

Thematic Domain	Primary Search Terms	Secondary/Combination Terms
Computational Thinking and Teacher Education	“computational thinking” OR “CT education” OR “algorithmic thinking”	AND (“teacher education” OR “preservice teacher” OR “pre-service teacher” OR “initial teacher training”)
Generative AI and Teacher Education	“generative AI” OR “generative artificial intelligence” OR “ChatGPT” OR “large language model” OR “prompt engineering”	AND (“teacher education” OR “preservice teacher” OR “higher education” OR “learning design”)
Psychology of Engagement	“self-efficacy” OR “cognitive load” OR “technology anxiety” OR “affective engagement” OR “professional identity”	AND (“computational thinking” OR “artificial intelligence” OR “emerging technology” OR “teacher education”)

## Data Availability

No new data were created or analyzed in this study.

## Supplementary Materials

The following supporting information can be downloaded at https://www.mdpi.com/article/10.3390/bs16040575/s1: Overview of studies.
